# Directional Construction of a Synthetic Microbial Consortium for Shanxi Aged Vinegar: Metabolic Kinetics and Accelerated Formation of Major Volatile Compounds

**DOI:** 10.3390/foods15183286

**Published:** 2026-09-17

**Authors:** Xuefeng Zhu, Limin Cui, Xianxian Luo, Fanfan Lang, Yu Zheng, Zhiqiang Nie

**Affiliations:** 1 Shanxi Provincial Key Laboratory for Vinegar Fermentation Science and Engineering, Key Laboratory of Special Sorghum Breeding and Processing (Shanxi), Ministry of Agriculture and Rural Affairs, Shanxi Zilin Flavoring and Food Co., Ltd., Taiyuan 030400, China; 2 Biomedicine and Health Laboratory in Shanxi Province, Key Laboratory of Chemical Biology and Molecular Engineering, Ministry of Education, Shanxi Key Laboratory of Bioengineering, Institute of Biotechnology, Shanxi University, Taiyuan 030006, China; 3 Shanxi Songjiagou Functional Food Co., Ltd., Xinzhou 036300, China; 4 State Key Laboratory of Bio-Based Fiber Materials, School of Biological Engineering, Tianjin University of Science and Technology, Tianjin 300457, China

**Keywords:** Shanxi aged vinegar, culturable microorganisms, synthetic microbial consortium, metabolic kinetics, volatile compounds, semi-quantitative HS-SPME-GC-MS

## Abstract

The *Daqu* used in the alcoholic fermentation of Shanxi aged vinegar (SAV) is technologically related to the medium-temperature *Daqu* used for Baijiu production, but variation in vinegar *Daqu* quality can lead to unstable saccharification, alcoholic fermentation and aroma formation. We therefore hypothesized that functional microorganisms selected from Light-Flavor Baijiu *Daqu* could be combined with isolates from vinegar fermentation to improve the alcoholic fermentation stage of SAV, and that a defined consortium would provide a controllable system in which to test this hypothesis. Twenty culturable microorganisms were isolated from *Daqu* and *Jiupei* (fermented grains). Six were preselected on the basis of taxonomic identity, fermentation-related function and intended functional complementarity, namely *Saccharomyces cerevisiae*, *Pichia kudriavzevii*, *Saccharomycopsis fibuligera*, *Lactiplantibacillus plantarum*, *Lentilactobacillus buchneri* and *Ligilactobacillus amylovorus*; their growth, stress tolerance, ethanol production and acid production were then characterized to assess their suitability for consortium construction. Headspace solid-phase microextraction coupled with gas chromatography–mass spectrometry (HS-SPME-GC-MS) was used to profile the volatile compounds produced during fermentation of the resulting synthetic microbial consortium (SMC) in a liquid sorghum-juice medium and to compare them with those of industrial alcoholic fermentation (IAF) of fermented grains. All experiments used three independent biological replicates, and concentrations are reported as semi-quantitative 2-octanol equivalents. Several major volatile compounds accumulated earlier in the SMC than in the IAF system: ethanol (0.741 mg/mL) and ethyl acetate (0.331 mg/mL) both peaked on day 3, and the day-3 ethyl acetate concentration in the SMC already exceeded the maximum value reached in the IAF system on day 7 (0.238 mg/mL). The SMC nevertheless produced fewer volatile compounds (30) than the IAF system (45) and lacked long-chain fatty acid ethyl esters, whereas multivariate analysis indicated that its volatile profile became progressively more uniform during the mid-to-late fermentation stages. Because the two systems differ in substrate composition, physical structure and mass transfer, these findings should not be read as evidence that the SMC outperforms industrial solid-state fermentation. Within that constraint, the SMC reproduced selected metabolic features of SAV alcoholic fermentation in a liquid model system and reached maximum concentrations of several major volatile compounds earlier than the industrial system examined, offering a starting point for the rational design of defined starter consortia for traditional vinegar production.

## 1. Introduction

As one of China’s four famous vinegars, Shanxi aged vinegar (SAV) owes its characteristic flavor and quality primarily to the traditional solid-state fermentation (SSF) process, in which the resident microbiota and its metabolic pathways determine both acid yield and aroma formation [1]. Alcoholic fermentation (AF) is the first and most critical step of the SAV production cycle, establishing the flavor precursors and determining the acid-production efficiency, and it relies on the natural microbial communities present in the environment. The aromatic complexity of SAV arises from the metabolic complementarity of this multispecies community. For example, *Saccharomyces cerevisiae* contributes primarily to ethanol formation and to the production of fermentation-derived volatile metabolites, whereas non-Saccharomyces yeasts such as *Pichia kudriavzevii* can contribute to ester-related aroma formation. *Saccharomycopsis fibuligera* may facilitate the release of fermentable substrates and flavor precursors through its hydrolytic activities, while lactic acid bacteria, including *Lactiplantibacillus plantarum* and *Lentilactobacillus buchneri*, contribute organic acids and other metabolites that further shape the overall aroma profile. However, the succession of natural microbial communities is highly susceptible to seasonal and environmental fluctuations. This makes the fermentation process difficult to regulate, leading to discrepancies in product quality between batches [2,3]. Synthetic microbial consortia with defined structure and controllable function have accordingly become an active focus of food biotechnology [4].

Research has therefore focused on identifying functional microorganisms and on regulating microbial interactions in traditional fermented foods. Studies of Chinese cereal-based fermentations have shown that *Saccharomyces cerevisiae* is a major contributor to ethanol production, whereas non-Saccharomyces yeasts can participate in aroma formation and substrate utilization [5]. Lactic acid bacteria (LAB) contribute organic acids and other metabolites and can interact with yeasts to modify metabolic fluxes and flavor profiles [6,7]. In addition, fortified inoculation with indigenous functional microorganisms has been reported as a potential strategy for improving the controllability and metabolic performance of traditional solid-state fermentation [8]. Flavor formation is therefore not attributable to a single dominant microorganism; it results from metabolic cooperation and competition among microorganisms that occupy complementary ecological and metabolic niches [9,10].

Defined synthetic microbial consortia (SMCs), in which selected microorganisms with complementary metabolic functions are rationally combined, have emerged as a promising strategy for improving the controllability and reproducibility of food fermentation [11]. Compared with spontaneous communities, SMCs reduce complexity while retaining selected functions such as saccharification, ethanol production, acid production and aroma formation. Nevertheless, most previous studies on traditional cereal fermentation have focused on microbial community characterization, individual functional strains or fortified inoculation. The rational construction of a defined multispecies consortium specifically for the alcoholic fermentation stage of SAV, together with a systematic evaluation of its fermentation kinetics and volatile-metabolite formation relative to an industrial fermentation system, remains insufficiently explored. Closing this gap will show whether core fermentation functions can be reconstructed with a simplified community and will support the development of more controllable, standardized fermentation processes [12,13].

Therefore, the objective of this study was to construct a function-oriented synthetic microbial consortium for the alcoholic fermentation stage of SAV and to evaluate its fermentation performance and flavor-forming capacity. Functional microorganisms isolated from *Daqu* and *Jiupei* were screened according to their physiological and fermentation-related characteristics and assembled into a six-strain consortium comprising yeasts and LAB. The fermentation kinetics and the dynamic accumulation of volatile compounds produced by the SMC were subsequently characterized and compared with those of industrial alcoholic fermentation (IAF). We thereby assessed the potential of a defined consortium to reproduce and accelerate selected metabolic functions of SAV alcoholic fermentation in a liquid model system while recognizing that compositional and process-related differences between the model system and the industrial solid-state process constrain direct comparison. The work was conceived as a step toward controllable and standardized microbial starters for traditional vinegar production.

## 2. Materials and Methods

### 2.1. Sample Collection

In June 2023, samples of *Daqu* (a traditional solid-state fermentation starter for Baijiu) and *Jiupei* (fermented grains) were collected from Shanxi Zilin Flavoring and Food Co., Ltd. Samples of *Jiupei* were collected on days 1, 3, 5 and 7 of the alcoholic fermentation process. The fermented-grain samples were withdrawn from routine industrial production rather than from experimentally manipulated fermentations and therefore represent a single production campaign; this is acknowledged as a limitation in Section 4. At each sampling time, five spatial subsamples were collected from each fermentation replicate using a five-point sampling strategy and combined to obtain one representative composite sample for that biological replicate. Thus, three independent biological samples (*n* = 3) were obtained at each fermentation time point. The samples were then sealed within sterile bags and transported to the laboratory in ice-cooled containers. Upon arrival, 100 mg aliquots were stored at −20 °C for immediate use, and the remaining samples were stored at −80 °C.

### 2.2. Isolation and Purification of Culturable Microorganisms

Samples were suspended in 18 mL of sterile saline, shaken at 30 °C and 200 rpm for 30 min, and subsequently centrifuged at 12,000× *g* for 10 min. The suspension was then serially diluted and spread onto LB agar (for bacteria), MRS agar (for lactic acid bacteria, LAB), and YPD agar (for yeasts and molds) (Sangon Biotech Co., Ltd., Shanghai, China). The plates were incubated at 30 °C (bacteria and fungi) or 37 °C (LAB) for 48 h. Colonies differing in morphology (size, color, margin and surface texture) were selected for further analysis, purified by repeated streaking and preserved in 20% (*v*/*v*) glycerol at −80 °C.

Morphological characterization was performed according to Bergey’s Manual of Systematic Bacteriology and General Mycology. Gram staining was performed on bacterial isolates and examined under 100× oil immersion microscopy. The yeast cells were subjected to staining with methylene blue, while filamentous fungal hyphae were examined following staining with carbol cotton blue.

### 2.3. Molecular Identification

Genomic DNA was extracted using bacterial and fungal genomic DNA extraction kits (Tiangen, Beijing, China) in accordance with the manufacturer’s instructions. The 16S rRNA gene was amplified using the primers 27F (5′-AGAGTTTGATCCTGGCTCAG-3′) and 1492R (5′-GGTTACCTTGTTACGACTT-3′). The internal transcribed spacer (ITS) region was amplified using the primers ITS1 (5′-TCCGTAGGTGAACCTGCGG-3′) and ITS4 (5′-TCCTCCGCTTATTGATATGC-3′). Purification and Sanger sequencing of the PCR products were conducted by Sangon Biotech (Shanghai, China). Sequences were compared against the NCBI GenBank database using BLASTn (https://blast.ncbi.nlm.nih.gov/Blast.cgi), and species assignment was based on ≥98% sequence identity; query coverage was recorded for every assignment. GenBank accession numbers, the closest related type strains, sequence identity and query coverage for all 20 isolates are provided in Supplementary Table S2.

### 2.4. Physiological Characterization of Selected Strains

#### 2.4.1. Growth Curve Analysis

Single colonies of each selected strain were inoculated into 5 mL of the corresponding liquid medium and cultured for 24 h. The activated cultures were then inoculated (1%, *v*/*v*) into 50 mL of fresh medium, and growth was monitored by measuring OD600 every 2 h for 48 h using an automatic growth curve analyzer (Bioscreen C, Oy Growth Curves Ab Ltd., Helsinki, Finland). Yeasts were cultivated at 30 °C and LAB were cultured at 37 °C. Three independently inoculated cultures were analyzed per strain (*n* = 3).

#### 2.4.2. Ethanol Tolerance Assay

Activated yeast cultures were inoculated (1%, *v*/*v*) into 5 mL of YPD broth containing ethanol at final concentrations of 3%, 5%, 7%, 9%, 11%, 13%, and 15% (*v*/*v*). Each gradient was prepared volumetrically by adding absolute ethanol to the medium to reach the designated final concentration. LAB were tested using the same ethanol gradient, with MRS broth as the basal medium. All treatments were performed in triplicate. Following static incubation at the respective optimal temperatures for 24 h, OD600 was measured using a spectrophotometer (UV-2600i, Shimadzu, Kyoto, Japan).

#### 2.4.3. Acetic Acid Tolerance Assay (Yeasts)

Activated yeast cultures were inoculated (1%, *v*/*v*) into 5 mL YPD broth, which had been supplemented with acetic acid at concentrations of 0, 2, 5, 8, 11, 14, 17, and 20 g/L. Cultures were incubated statically at 30 °C for 24 h, with three replicates per treatment. OD600 was then measured as described in Section 2.4.2, Ethanol Tolerance Assay.

#### 2.4.4. Acid Production by Lactic Acid Bacteria

Activated LAB cultures were then inoculated (1%, *v*/*v*) into 100 mL of MRS broth containing 4% (*v*/*v*) ethanol and incubated statically at 37 °C in triplicate. Samples were collected aseptically at 24 h intervals, and the total acid content was determined by acid–base titration using 0.1 M NaOH with phenolphthalein as the indicator. The results were expressed as lactic acid equivalents (g/L).

#### 2.4.5. Ethanol Production by Yeasts

Yeast seed cultures were inoculated into the fermentation medium and incubated at 30 °C with shaking at 200 rpm for 7 days. Samples were collected daily, and the ethanol content was measured using an alcohol meter. An aliquot of fermentation broth was applied to the measurement area of the alcohol meter, and the ethanol content was recorded once the reading had stabilized. The maximum ethanol content measured during the 7-day fermentation period was used to evaluate the ethanol-producing capacity of each yeast strain. All measurements were performed in triplicate.

### 2.5. Construction of the Synthetic Microbial Consortium (SMC) and Fermentation

On the basis of the isolation results, six strains were selected for construction of the SMC: *Saccharomyces cerevisiae*, *Pichia kudriavzevii*, *Saccharomycopsis fibuligera*, *Lactiplantibacillus plantarum*, *Lentilactobacillus buchneri* and *Ligilactobacillus amylovorus*. The 20 isolates were not ranked against one another by a single quantitative scoring procedure. Instead, the initial SMC was deliberately designed as a simplified yeast–LAB consortium targeting the alcoholic fermentation stage of SAV: three yeasts and three LAB strains were preselected on the basis of taxonomic identity, fermentation-related function and intended functional complementarity, and their growth, stress tolerance, ethanol production and acid-production characteristics were then evaluated (Section 3.2) to assess their suitability for consortium construction. The remaining isolates were not regarded as functionally irrelevant but were excluded from this initial consortium to limit community complexity; the criteria applied to each of the six selected strains are summarized in Section 4. Each strain was individually activated in the corresponding medium and cultured to an OD600 of 1.5 before inoculation. Yeast strains were cultivated in YPD broth at 30 °C, whereas LAB strains were cultivated in MRS broth at 37 °C. The standardized seed cultures were mixed at an equal volumetric ratio (1:1:1:1:1:1, *v*/*v*) and co-inoculated (2%, *v*/*v* each) into 100 mL of sorghum juice medium in 250 mL Erlenmeyer flasks, giving a total inoculation level of 12% (*v*/*v*). Standardizing each seed culture to the same OD600 avoided deliberate weighting toward any individual strain at inoculation. Viable cell counts were not determined for the individual strains, however, so the inoculation ratio is defined on a volumetric rather than a cell-number basis, and the actual proportions of the six strains in terms of cell number at the time of inoculation may differ from 1:1. Because alternative inoculation ratios were not evaluated, the 1:1:1:1:1:1 ratio is presented as a starting design and is not claimed to be optimal. The flasks were incubated at 30 °C with shaking at 200 rpm. Three independently inoculated fermentation flasks were prepared and treated as independent biological replicates (*n* = 3). At each designated fermentation time point, samples were collected separately from each of the three flasks and processed independently for subsequent analyses. Samples (3 mL) were collected at 24, 48, 72, 96, 120 and 168 h of fermentation; the 24, 72, 120 and 168 h samples correspond to days 1, 3, 5 and 7, respectively. The supernatant was collected after centrifugation at 12,000× *g* for 5 min, flash-frozen in liquid nitrogen for 15 min, and stored at −80 °C for subsequent analysis.

Industrial alcoholic fermentation (IAF) samples were obtained from routine production at the Shanxi Zilin Flavoring and Food Co., Ltd. facility and were not experimentally manipulated. The IAF system is a solid-state process in which the sorghum-based substrate is saccharified and fermented by the resident *Daqu*-derived microbiota under the standard plant protocol. The IAF process parameters, including substrate composition, *Daqu* addition rate, temperature profile, fermentation duration and vessel configuration, are summarized in Supplementary Table S4. Because the IAF samples originated from a single production campaign, they are treated as process replicates within one batch rather than as independent production batches; this is acknowledged as a limitation in Section 4.

### 2.6. HS-SPME-GC-MS Analysis of Volatile Compounds

#### 2.6.1. Sample Preparation

For the *Jiupei* samples, 25 g of fermented grain collected on days 1, 3, 5 and 7 were mixed with 50 mL of ultrapure water and subjected to ultrasonic extraction in an ice bath for 30 min. The mixture was then centrifuged at 10,000× *g* and 4 °C for 20 min. An aliquot of 1 mL of the resulting extract was transferred to a 20 mL headspace vial. Subsequently, 1 g of NaCl, 1 μL of internal standard (2-octanol, 0.16 mg/mL in methanol) and 3 mL of ultrapure water were added. The vial was then immediately sealed with a PTFE–silicone septum.

For SMC fermentation broth, 1 mL of culture filtrate (passed through a 0.22 μm membrane filter) was placed in a 20 mL headspace vial, and 1 g of NaCl, 1 μL of 2-octanol (0.16 mg/mL), and 3 mL of ultrapure water were added before sealing.

For both systems, the three biological replicates were prepared and analyzed independently. Each biological sample was subjected to three replicate gas chromatography–mass spectrometry (GC-MS) measurements as technical replicates, and the three measurements were averaged before statistical analysis so that they were not treated as independent biological observations. The two systems were prepared in different physical forms: a clarified liquid culture for the SMC and as an aqueous extract of solid fermented grains for the IAF system. Section 2.6.4 discusses the consequences of this difference for quantitative comparison.

#### 2.6.2. SPME Procedure

The SPME fiber (50/30 μm DVB/CAR/PDMS, Supelco, Bellefonte, PA, USA) was conditioned at 270 °C for 30 min prior to use. The fiber was exposed to the headspace of the vial at 55 °C for 30 min with agitation, followed by a 5 min equilibration period. The fiber was then desorbed in the GC injection port at 250 °C for 5 min.

#### 2.6.3. GC-MS Conditions

Gas chromatography–mass spectrometry (GC-MS) analysis was performed using a Shimadzu QP2020 NX system (Shimadzu, Kyoto, Japan) equipped with a DB-WAX capillary column (30 m × 0.25 mm i.d., 0.25 μm film thickness; Agilent Technologies, Santa Clara, CA, USA). The injection port was operated in splitless mode at 250 °C. The oven temperature program was as follows: initial temperature of 35 °C held for 1 min, ramped to 150 °C at 5 °C/min, then to 230 °C at 10 °C/min, and held at 230 °C for 5 min. High-purity helium (≥99.999%) was used as the carrier gas, with a constant flow rate of 1.0 mL/min. The mass spectrometer was operated in electron impact (EI) mode at 70 eV, with the ion source at 250 °C and the transfer line temperature at 250 °C. Mass spectra were recorded in full-scan mode over a mass range of m/z 33–550.

#### 2.6.4. Identification and Quantification

Volatile compounds were identified by comparing their mass spectra with the NIST 17 library (National Institute of Standards and Technology, Gaithersburg, MD, USA) and by matching experimental retention indices (RI), calculated from the retention times of an n-alkane series analyzed under the same chromatographic program, with values reported in the literature for columns of the same or equivalent stationary phase. A compound was accepted only when the mass spectral match and the experimental RI were both consistent with the corresponding reference values. Because authentic standards were not analyzed in this study, no compound was confirmed at the highest level of identification confidence; all assignments are therefore reported as tentative identifications, and the experimental RI, the literature RI and the spectral match quality for the compounds relevant to the conclusions are listed in Supplementary Table S3. Quantification was performed using the internal standard method, with peak areas normalized to that of 2-octanol. Contents are reported as semi-quantitative concentrations expressed as 2-octanol equivalents (mg/mL) and not as absolute concentrations. Headspace SPME response is strongly matrix-dependent. The SMC samples were analyzed as a clarified liquid culture, whereas the IAF samples were analyzed as an aqueous extract of solid fermented grains, so the two preparations differ in phase composition, solute content and partitioning behavior. Concentrations obtained for the two systems are therefore not strictly comparable in absolute terms, and the comparison is interpreted only in terms of temporal trends within each system and of rank-order differences between systems.

### 2.7. Statistical Analysis

All experiments were performed with three independent biological replicates (*n* = 3). Three independently inoculated flasks were used for SMC fermentation, and three independent fermentation replicates were used for IAF; for each IAF replicate, five spatial subsamples were pooled at each sampling time. Each biological sample was analyzed three times by GC-MS as technical replicates, and the three measurements were averaged before statistical analysis so that they were not treated as independent observations. Data are presented as mean ± SD. Differences in volatile-compound concentrations between the two fermentation systems (SMC, IAF) and among sampling times (days 1, 3, 5 and 7) were evaluated by two-way analysis of variance (ANOVA) with fermentation system and sampling time as fixed factors, followed by Tukey’s honestly significant difference (HSD) test for pairwise comparisons; *p* values were adjusted for multiple comparisons and significance was set at *p* < 0.05. This replaced the independent-samples *t*-tests used in the previous version of the manuscript, which did not control the family-wise error rate across the multiple compounds, strains, concentrations and time points examined. Principal component analysis (PCA) was performed using SIMCA 14.1 (Umetrics, Umeå, Sweden) with Pareto scaling; model quality was summarized by the cumulative explained variance R2X(cum) and the cross-validated predictive ability Q2(cum) obtained by seven-fold cross-validation, and the risk of overfitting was assessed by permutation testing. Hierarchical clustering heatmaps were generated using the pheatmap package in R (R Foundation for Statistical Computing, version 4.6.1, Vienna, Austria). All figures were prepared using Origin 2025 (OriginLab, Northampton, MA, USA) or the ggplot2 package in R. Error bars in all quantitative figures represent the standard deviation of three independent biological replicates.

## 3. Results

### 3.1. Isolation and Identification of Functional Microorganisms

A total of 20 culturable microbial strains were successfully isolated from the *Daqu* and *Jiupei*. Morphological observation revealed distinct colony characteristics. Four strains were identified from *Daqu*. *Saccharomycopsis fibuligera* (DQ-2) formed round, white villous colonies with firm medium attachment and oval spores. *Aspergillus flavus* (DQ-5) exhibited irregular colonies with mycelia and globose conidia. The yeast strain *Saccharomyces cerevisiae* (DQ-6) produced smooth, milky white colonies with oval budding cells. In contrast, the yeast strain *Pichia kudriavzevii* (DQ-10) formed colonies distinguished by a yellowish-brown hue and an elliptical cell morphology.

**Figure 1:**
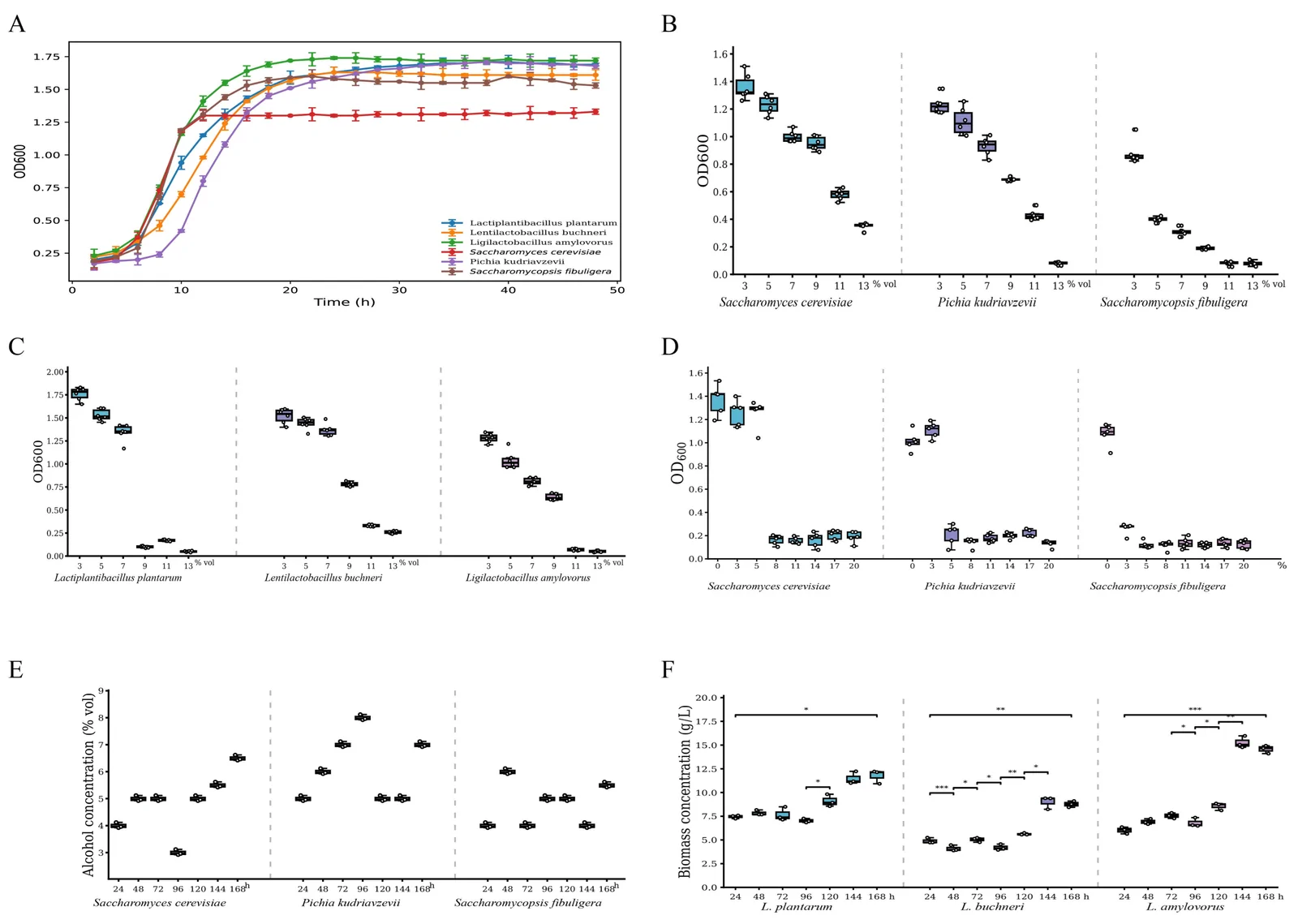
Physiological characterization of the six strains selected for SMC construction. (**A**) Growth curves of the six microbial strains. (**B**) Ethanol tolerance of yeasts. (**C**) Ethanol tolerance of lactic acid bacteria (LAB). (**D**) Acetic acid tolerance of yeasts. (**E**) Ethanol production by yeasts. (**F**) Acid production by LAB. Data are presented as mean ± SD of three independent biological replicates (*n* = 3); where not visible, error bars fall within the symbols. Statistical significance is indicated by asterisks: one asterisk (*) denotes *p* < 0.05, two asterisks (**) denote *p* < 0.01, and three asterisks (***) denote *p* < 0.001; ns indicates not significant. *p* values were determined by *t*-test.

A total of 16 strains were isolated and identified from *Jiupei*, predominantly lactic acid bacteria (LAB) and *Bacillus* species. The LAB isolates, including *Lactiplantibacillus plantarum* (JP-08), *Lentilactobacillus buchneri* (JP-10), *Ligilactobacillus amylovorus* (JP-90), *Levilactobacillus brevis* (JP-56) and *Limosilactobacillus fermentum* (JP-68), typically formed small, smooth, milky white or yellowish colonies and appeared as short rods or ellipsoids under microscopy. In addition, several *Bacillus* species were identified, including *Bacillus subtilis*, *Bacillus licheniformis* and *Bacillus megaterium*; the latter two produced larger, irregular and rough colonies composed of long rods. *Bacillus cereus* (JP-40) and *Aspergillus flavus* (DQ-5) were also recovered; the safety implications of these isolates are addressed in Section 4. Condensed morphological descriptions are given in Table 1, and the complete colony and microscopic characteristics are reported in Supplementary Table S1. Phylogenetic analysis, performed separately for the bacterial 16S rRNA gene sequences and for the fungal and yeast ITS sequences as described in Section 2.3, confirmed these identifications and revealed close evolutionary relationships among the LAB isolates and between *S. cerevisiae* and *S. fibuligera* (Figure 1).

### 3.2. Physiological and Biochemical Characterization of the Selected Strains

Six strains were selected from the isolates for physiological characterization (*S. cerevisiae*, *P. kudriavzevii*, *S. fibuligera*, *L. plantarum*, *L. buchneri* and *L. amylovorus*) to assess their suitability for the construction of the SMC (Figure 1).

Growth curve analysis demonstrated that *L. plantarum*, *L. buchneri*, *L. amylovorus* and *P. kudriavzevii* entered the logarithmic phase at approximately 4 h and reached the stationary phase at 18 h, whereas *S. cerevisiae* entered the stationary phase earlier, at 12 h. Ethanol tolerance tests indicated that *S. cerevisiae* exhibited the strongest tolerance, maintaining slight growth at 13% (*v*/*v*) ethanol. Growth of *P. kudriavzevii* and *S. fibuligera* was severely inhibited when the ethanol concentration exceeded 9% and 5% (*v*/*v*), respectively. Among the LAB, *L. plantarum* was relatively sensitive, with a marked decline in growth at 9% (*v*/*v*) ethanol, and all three LAB strains ceased growth at 13% (*v*/*v*) ethanol. With respect to acetic acid tolerance, *S. cerevisiae* and *P. kudriavzevii* grew normally at acetic acid concentrations of up to 2 g/L, but their growth was markedly restricted at concentrations ≥ 5 g/L. *Ligilactobacillus amylovorus* showed the highest acid production, reaching a maximum total acid concentration of 15.42 g/L at 168 h. Acid production by the three LAB strains increased continuously until 168 h and then stabilized. Finally, ethanol production by the yeast strains was monitored over 7 days. The ethanol content of the yeast fermentation broths remained relatively stable at approximately 5% (*v*/*v*), with *P. kudriavzevii* reaching a peak of 7%. These physiological characteristics supported the use of the six strains in subsequent mixed fermentation. All assays were performed with three independent biological replicates (*n* = 3), and values are reported as mean ± SD.

### 3.3. Dynamic Changes in Volatile Compounds During Fermentation by the Synthetic Microbial Consortium

**Figure 2:**
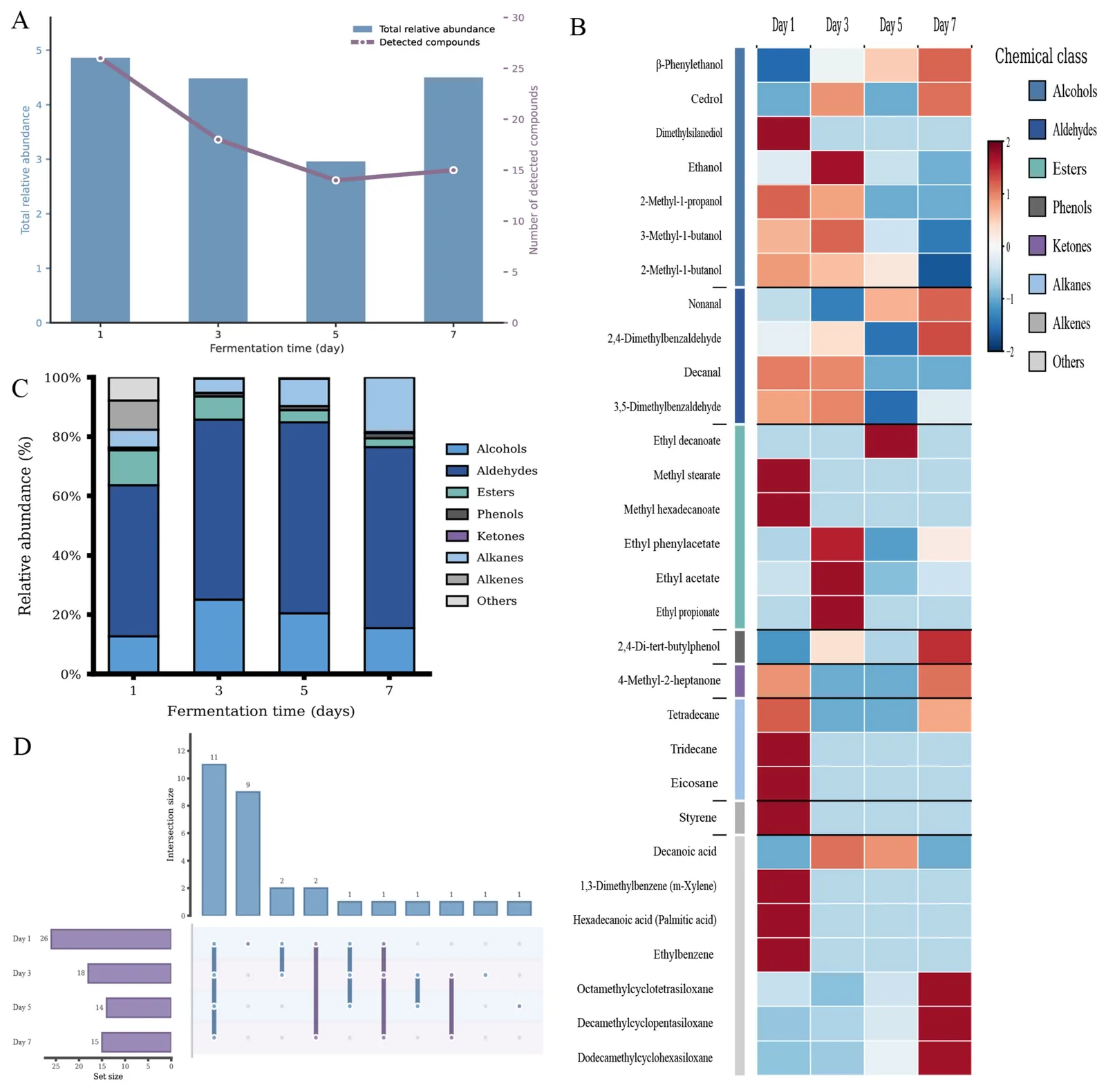
Dynamic evolution of volatile compounds during alcoholic fermentation by the SMC. (**A**) Changes in total volatile abundance and in the number of detected volatile compounds. (**B**) Heatmap showing the dynamic abundance patterns of all detected volatile compounds. (**C**) Temporal changes in the relative abundance of different classes of volatile compounds. (**D**) UpSet plot showing the shared and unique volatile compounds among fermentation stages. Concentrations are semi-quantitative 2-octanol equivalents. Values are the mean ± SD of three independent biological replicates (*n* = 3).

During fermentation by the SMC, total volatile abundance initially decreased and then rebounded (Figure 2A). Total volatile abundance on days 1, 3, 5 and 7 was 4.86, 4.48, 2.96 and 4.50 mg/mL, respectively. Relative to day 1, abundance decreased by 7.70% on day 3 and 39.06% on day 5 and rebounded to 4.50 mg/mL on day 7. The number of volatile compounds detected decreased from 26 on day 1 to 15 on day 7, indicating that the volatile profile narrowed during fermentation and became dominated by a few compounds.

**Figure 3:**
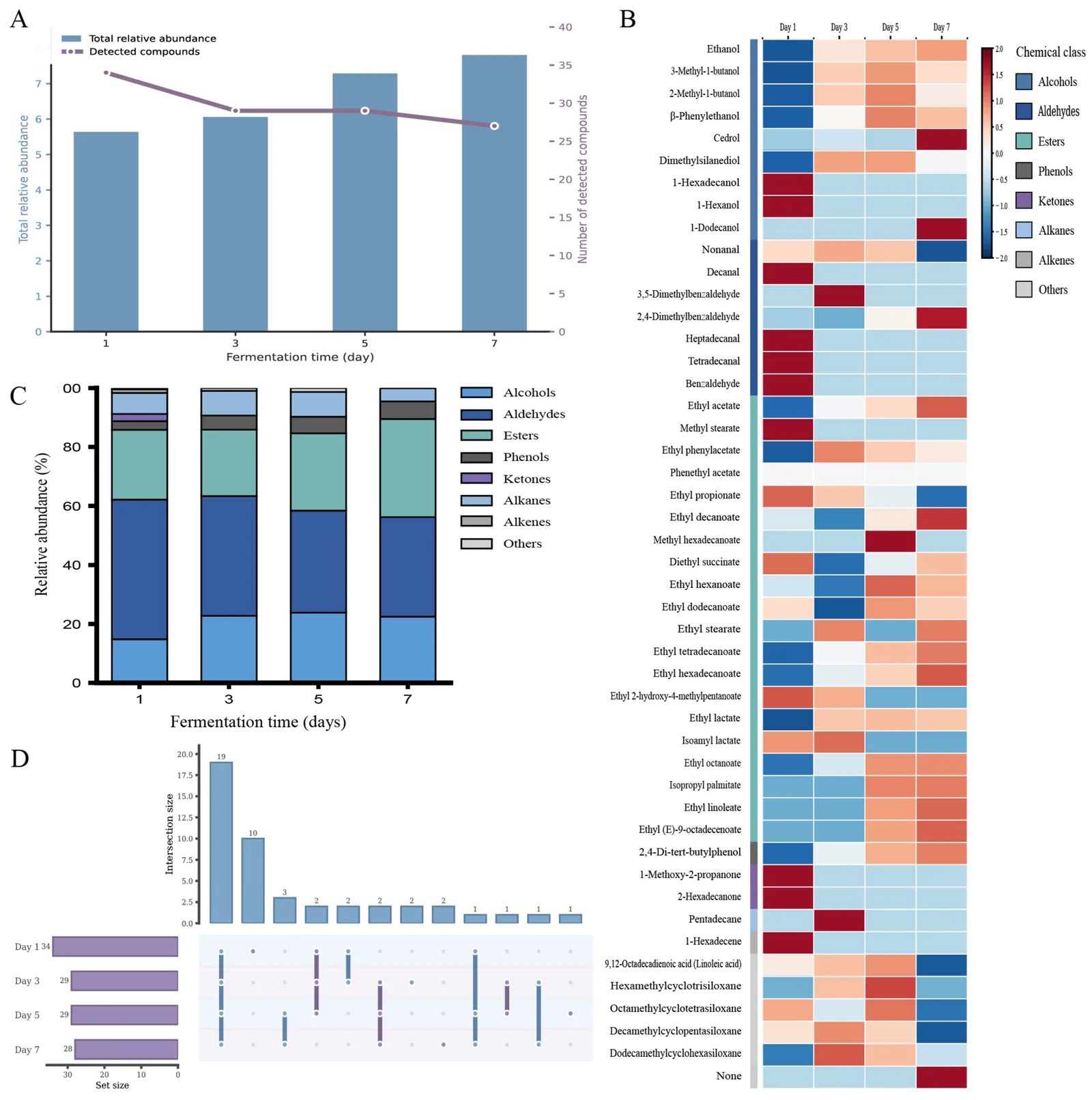
Dynamic evolution of volatile compounds during the industrial alcohol fermentation (IAF) process. (**A**) Changes in total volatile abundance and in the number of detected compounds during fermentation. (**B**) Heatmap showing the abundance patterns of all detected volatile compounds across fermentation stages. (**C**) Relative abundance of different chemical classes of volatile compounds during fermentation. (**D**) UpSet plot illustrating the shared and unique volatile compounds detected at different fermentation stages. Concentrations are semi-quantitative 2-octanol equivalents. Values are the mean ± SD of three independent biological replicates (*n* = 3).

The heatmap shows distinct time-dependent patterns among individual metabolites (Figure 3B). 2,4-Dimethylbenzaldehyde was relatively abundant at all four time points (2.22, 2.37, 1.87 and 2.69 mg/mL, respectively) and was the most abundant putatively identified volatile component throughout SMC fermentation. As noted in Section 2.6.4, this compound was assigned by mass spectral and retention index matching only and was not confirmed with an authentic standard; its identity and concentration should therefore be interpreted with caution. Ethanol peaked at 0.741 mg/mL on day 3 and then declined to 0.271 mg/mL on day 5 and 0.212 mg/mL on day 7. As shown in Figure 2B, ethyl acetate increased to a maximum of 0.331 mg/mL on day 3 before decreasing to 0.0979 mg/mL on day 5 and 0.1206 mg/mL on day 7. The concentration of 3-methyl-1-butanol decreased steadily from 0.181 mg/mL on day 3 to 0.0372 mg/mL on day 7. In contrast, β-phenethyl alcohol accumulated continuously from 0.0380 mg/mL on day 1 to 0.440 mg/mL on day 7. The concentration of 3,5-dimethylbenzaldehyde increased to 0.317 mg/mL on day 3, was not detected on day 5 and was 0.0181 mg/mL on day 7. These results indicate that this compound was formed primarily during the early stages of fermentation.

The relative composition of the chemical classes shows that aldehydes dominated throughout fermentation (Figure 2C), with their proportion increasing from 50.98% on day 1 to 61.01% on day 7. The proportion of alcohols increased to 25.08% on day 3 and then decreased to 15.46% on day 7. The proportion of esters decreased consistently from 11.79% on day 1 to 7.86% on day 3, 4.11% on day 5 and 2.97% on day 7, consistent with the decline in ethyl acetate and in specific fatty acid esters. The proportion of phenols increased gradually from 0.69% on day 1 to 1.79% on day 7. The proportion of alkanes rose to 18.49% on day 7, far above the values recorded at the three preceding time points. Alkenes were detected only on day 1 (9.86% of the total) and were not detected thereafter.

The UpSet analysis resolved 10 non-empty intersections (Figure 2D). Eleven volatile compounds were detected at all four time points. Eight of these were the dominant components: ethanol, 3-methyl-1-butanol, β-phenethyl alcohol, nonanal, 2,4-dimethylbenzaldehyde, ethyl acetate, ethyl phenylacetate and 2,4-di-tert-butylphenol. Day 1 had nine unique compounds, more than at any other stage, whereas days 3, 5 and 7 each had a single unique compound. Compounds common to all four time points accounted for 73.3% of the compounds detected on day 7, indicating that the late fermentation stage consisted mainly of persistent compounds, whereas early-stage compounds such as styrene, methyl hexadecanoate, ethylbenzene and m-xylene disappeared in subsequent stages.

During SMC fermentation, therefore, both the total abundance and the diversity of volatile compounds decreased during the middle phase; by day 7 the total abundance had recovered, but the diversity had not. The changes were characterized by the sustained predominance of aldehydes, a gradual decline in the proportion of esters, the continuous accumulation of β-phenethyl alcohol and the concentration of key volatile compounds during the late fermentation phase.

### 3.4. Dynamic Changes in Volatile Compounds During Industrial Alcohol Fermentation (IAF)

As shown in Figure 3A, total volatile abundance increased continuously during the IAF process. Total volatile abundance on days 1, 3, 5 and 7 was 5.64, 6.06, 7.29 and 7.81 mg/mL, respectively, representing a 38.4% increase from day 1 to day 7. Conversely, the number of volatile compounds detected decreased gradually from 34 on day 1 to 28 on day 7, suggesting that the increase in volatile abundance was predominantly attributable to the accumulation of dominant compounds rather than to the continuous generation of new volatiles.

The heatmap shows distinct temporal patterns among individual metabolites (Figure 3B). Ethanol increased steadily throughout fermentation from 0.507 mg/mL on day 1 to 0.934 mg/mL on day 7. Concurrently, β-phenethyl alcohol increased continuously from 0.190 to 0.471 mg/mL. The concentrations of ethyl acetate, ethyl lactate, diethyl succinate and various ethyl esters of long-chain fatty acids increased markedly during the late stages of fermentation. In contrast, aldehydes such as 2,4-dimethylbenzaldehyde remained relatively stable throughout fermentation. Several compounds, including ketones and alkenes, were present during the initial stage but absent in subsequent stages, indicating that the volatile profile was continuously restructured throughout the process.

The composition of the volatile chemical classes also changed during fermentation (Figure 3C). Aldehydes remained the most prevalent class throughout, accounting for 47.3%, 40.5%, 34.6% and 33.7% of total volatile abundance on days 1, 3, 5 and 7, respectively. The proportion of esters increased gradually from 23.7% on day 1 to 33.3% on day 7, becoming the second-largest class in the late fermentation stage. Alcohols increased from 14.8% on day 1 to 22.5% on day 7, while phenols accumulated from 2.9% to 6.0%. Ketones and alkenes were detected only on day 1, whereas the relative abundance of alkanes remained between 5% and 8% throughout fermentation.

The UpSet analysis resolved 19 volatile compounds present at all four fermentation stages (Figure 3D), accounting for 67.9% of the compounds detected on day 7. Day 1 exhibited the highest number of stage-specific compounds (10), whereas days 5 and 7 each contained only a single unique compound. Only two or three compounds were shared between adjacent fermentation stages. These results indicate that the volatile profile gradually simplified during fermentation, while the primary aroma components were consistently retained and accumulated.

Total volatile abundance therefore increased continuously throughout the IAF process, whereas the number of detected compounds declined gradually. The accumulation of alcohols and esters, together with the sustained predominance of aldehydes, gave the later stages of fermentation a relatively stable volatile profile.

### 3.5. Comparison of Volatile Compounds in the SMC and Industrial Alcohol Fermentation (IAF)

**Figure 4:**
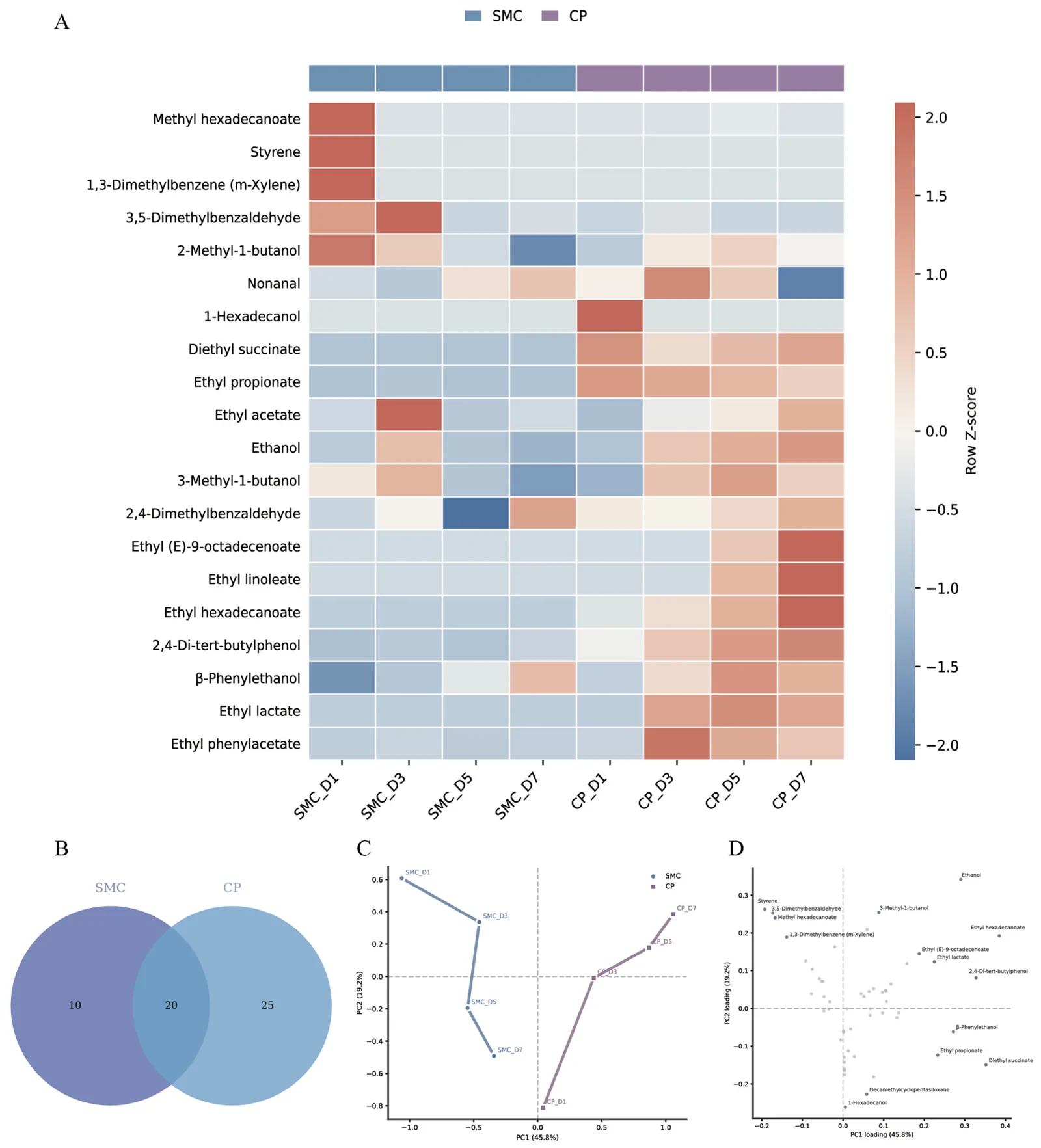
Comparative analysis of the volatile compounds produced by the SMC and by the traditional industrial alcoholic fermentation (IAF) during alcoholic fermentation. (**A**) Heatmap showing the relative abundance patterns of the major volatile compounds in the SMC and IAF at different fermentation stages. (**B**) Venn diagram showing the shared and unique volatile compounds detected in the two systems. (**C**) PCA score plot illustrating the overall differences in volatile profiles between the two systems during fermentation. (**D**) PCA loading plot showing the major volatile compounds contributing to sample discrimination. Concentrations are semi-quantitative 2-octanol equivalents (*n* = 3 independent biological replicates).

Figure 4A shows the abundance patterns of the 20 most abundant volatile compounds across the eight SMC and IAF samples. In the early stages, SMC samples were characterized by 3,5-dimethylbenzaldehyde, styrene, m-xylene and methyl hexadecanoate; the dominant constituents changed as fermentation progressed. In the middle and late stages of IAF, ethanol, β-phenethyl alcohol, diethyl succinate, ethyl lactate, ethyl hexadecanoate and ethyl esters of unsaturated fatty acids became the dominant components. Ethyl acetate peaked on day 3 in the SMC, whereas in the IAF system its concentration increased gradually over time. The identities of styrene, m-xylene and the dimethylbenzaldehyde isomers were not confirmed with authentic standards (Section 2.6.4); these compounds are therefore reported as putatively identified, and their occurrence is described without attributing to them a specific sensory role. With that qualification, the high-abundance volatiles in the SMC were concentrated in certain aldehydes and aromatic compounds, whereas the later stages of IAF showed a greater tendency toward the accumulation of alcohols and esters.

Venn analysis resolved 55 volatile compounds, of which 20 were common to both systems, accounting for 36.4% of the total (Figure 4B). Ten volatile compounds were specific to the SMC, while 25 were specific to the IAF system. The number of IAF-specific compounds (25) was approximately 2.5 times that of SMC-specific compounds (10), indicating that the traditional vinegar mash produced a richer array of volatile metabolites. The engineered consortium reduced volatile complexity but still retained some of the shared volatile components.

PCA further revealed the overall differences in the volatile profiles of the two fermentation systems (Figure 4C). The first two principal components accounted for 65.0% of the total variance, with PC1 and PC2 explaining 45.8% and 19.2%, respectively; the model was cross-validated as described in Section 2.7. All SMC samples were distributed along the negative axis of PC1, while IAF samples were concentrated along the positive axis, and the two groups were clearly separated, indicating that the engineered consortium and the traditional vinegar mash had distinct volatile profiles. Both systems continued to change throughout fermentation: SMC samples shifted predominantly along the negative PC2 axis from day 1 to day 7, whereas IAF samples shifted gradually toward positive PC1 and PC2, indicating that the two systems followed different trajectories of volatile succession.

PCA loadings revealed the contributions of individual volatile compounds to the separation of the two fermentation systems (Figure 4D). Ethanol, ethyl hexadecanoate, ethyl lactate, 2,4-di-tert-butylphenol, β-phenethyl alcohol, diethyl succinate, ethyl propionate and ethyl oleate lay along the positive direction of PC1 and were therefore the primary variables separating the IAF samples; styrene, 3,5-dimethylbenzaldehyde, m-xylene and methyl hexadecanoate were distributed mainly along the negative direction of PC1 and contributed strongly to the separation of the SMC samples. Ethanol had the largest loading, indicating that it contributed most to the discrimination between the two systems. The esters clustered along the positive direction of PC1, confirming that ester accumulation during the middle and late stages of IAF was the main source of divergence between the two systems.

The engineered consortium shared 20 of the 55 volatile compounds detected across the two systems, produced a markedly smaller number of volatiles than the IAF system and followed a distinct fermentation trajectory. The traditional vinegar mash was characterized by a more complex volatile composition and a stronger tendency toward ester accumulation, whereas the engineered consortium tended to form a volatile profile dominated by aldehydes and certain higher alcohols. Because the two systems also differ in matrix and process configuration, this contrast reflects the combined effect of microbial composition and fermentation system rather than microbial composition alone.

## 4. Discussion

A total of 20 culturable microbial strains belonging to at least ten genera were isolated from Light-Flavor Baijiu *Daqu* and *Jiupei*. Six core functional strains were identified from these isolates, and a six-strain SMC was constructed using *Saccharomyces cerevisiae*, *Pichia kudriavzevii*, *Saccharomycopsis fibuligera*, *Lactiplantibacillus plantarum*, *Lentilactobacillus buchneri* and *Ligilactobacillus amylovorus*.

The six strains were selected to establish a simplified yeast–LAB consortium focusing on the alcoholic fermentation stage of SAV. The three yeasts were included to provide ethanol-production, fermentation-adaptation and potential substrate-utilization functions, whereas the three LAB strains were selected for their complementary acid-production characteristics. The remaining isolates were not considered functionally irrelevant but were excluded from the initial consortium to limit community complexity; in particular, although *Bacillus* spp. and filamentous fungi may contribute hydrolytic enzymes and flavor precursors during traditional solid-state fermentation, their incorporation would require additional functional and strain-level safety evaluation. Safety considerations were also taken into account when interpreting the potential application of the isolates. Although *Aspergillus flavus* and *Bacillus cereus* were recovered during culture-dependent isolation, they were not included in the SMC, because isolates belonging to these species may present strain-dependent toxigenic or pathogenic risks and would require dedicated safety assessment before food application. In addition, although *Pichia kudriavzevii* was included in the present experimental consortium because of its fermentation-related characteristics, its intended use in food fermentation would likewise require strain-level safety evaluation before practical application. The present work should therefore be regarded as a fermentation-model study rather than as evidence of food-safety qualification of the individual strains.

The SMC reached a peak ethyl acetate concentration of 0.331 mg/mL by day 3 of fermentation, whereas the maximum concentration observed in the traditional solid-state fermented grains (*Jiupei*) on day 7 was 0.238 mg/mL. This difference should not be interpreted as evidence that the consortium is intrinsically more productive than the industrial process. The two systems differ in substrate composition, water activity, physical structure, oxygen transfer and sample preparation, and the volatile data are semi-quantitative (Section 2.6.4); a substantial part of the observed difference may therefore reflect matrix- and process-related effects rather than microbial metabolism alone. Within that constraint, the earlier accumulation of ethyl acetate is consistent with a redistribution of metabolic fluxes within the simplified system. In traditional solid-state fermented grains, community assembly of the functional microorganisms is complex, involving niche competition with non-core members and a prolonged adaptation period during which metabolic activity is constrained by interspecific competition. van Tatenhove-Pel et al. [9] demonstrated that microbial competition can reduce metabolic interaction distances to the low-micrometer range, suggesting that in a simplified consortium the close spatial proximity of functional strains may decrease metabolic diffusion loss. Jin et al. [14] reviewed microbial interaction mechanisms in traditional solid-state fermentation and highlighted that metabolite production in conventional multispecies systems is strongly influenced by community hierarchical structure. 

An important qualification follows from this comparison. The SMC was cultivated in a liquid medium, whereas the industrial process operates on solid fermented grains, so differences in substrate composition, water activity, oxygen transfer, mass transfer and sample preparation co-vary with the difference in microbial composition. The present design therefore does not allow effects attributable to microbial composition to be separated quantitatively from those attributable to the fermentation matrix. The results should be read as a comparison between two fermentation systems rather than as an isolated assessment of the metabolic capacity of the consortium, and a definitive attribution will require the consortium to be evaluated in a solid-state matrix.

At the esterification level, ethanol produced by yeasts and acetic acid generated by LAB condense to form ethyl acetate, a reaction catalyzed by alcohol acetyltransferase [15]. All three LAB strains examined in this study exhibited acid-producing capacity, with *L. amylovorus* accumulating 15.42 g/L of total acids within 168 h, thereby providing an acyl-donor pool for esterification. Deng et al. [5] reported that co-cultures of *P. kudriavzevii* and *S. cerevisiae* exhibited a cooperative response to organic acid stress, maintaining higher ethanol concentrations and cell viability in a medium containing 6 g/L lactic acid. The close spatial arrangement of yeasts and LAB within the SMC may favor a comparable interaction, in which yeast-derived ethanol and bacterium-derived organic acids become available within the same phase and time window. Because the present design did not include monocultures of the individual strains or simplified yeast–LAB combinations, however, the relative contributions of interspecific interaction and of the additive metabolism of the individual strains cannot be separated; for this reason the term synergistic is avoided here. Wu et al. [16] showed that microbial interspecific interactions can modify the flavor profile of Shanxi aged vinegar and that simplified communities can enhance specific metabolic outputs.

The enzyme-producing strain *Saccharomycopsis fibuligera* was included in the SMC for its extracellular hydrolytic capacity, serving as a non-*Daqu*-dependent saccharification driver. Mou et al. [17] reported that *S. fibuligera* strains isolated from traditional starter cultures exhibited potent amylase and protease activities and that their solid-state fermentation products contained a high proportion of flavor-active compounds, including β-phenylethanol and isoamyl acetate. In the liquid SMC system, the extracellular enzyme system of *S. fibuligera* may facilitate the breakdown of macromolecular starch from sorghum and barley, thereby alleviating the rate-limiting step of saccharification and indirectly contributing to the synthesis of the targeted metabolites. Li et al. [18] further characterized the enzyme-production profiles of indigenous microbial communities using metabolomics, supporting the metabolic contribution of this species to carbohydrate cleavage.

Despite reconstituting several characteristic compounds of SAV, including ethyl acetate, ethanol, phenylethanol and 2,4-dimethylbenzaldehyde, the SMC produced a markedly less diverse volatile profile than the traditional IAF process. Only four esters were detected in the SMC, whereas 15 were identified in the traditional system, and long-chain fatty acid ethyl esters such as ethyl octanoate, ethyl palmitate and diethyl succinate were not detected. This shortfall reflects the raw materials and the multiphase matrix of SAV fermentation and can be traced to three factors:

First, precursor pathways are missing. In traditional industrial alcoholic fermentation, raw materials such as sorghum and wheat bran provide abundant crude lipids and proteins. Concurrently, indigenous filamentous fungi (*Aspergillus* and *Rhizopus*) and *Bacillus* species possess extracellular enzyme systems, including lipases and proteases, that decompose raw materials to release long-chain fatty acids and amino acid precursors Song et al. [19] employed metatranscriptomic sequencing to characterize the core functional microbiota of solid-state fermentation and found that the expressed enzyme genes of *Bacillus* and *Aspergillus* dominated the carbohydrate hydrolysis and lipid metabolism pathways. Wang et al. [11] reached a similar conclusion, confirming that *Bacillus* is a key genus driving flavor metabolite production during the solid-state alcoholic fermentation (SSAF) stage. The simplified SMC, consisting exclusively of yeasts and LAB, lacks the macromolecular lipid-degrading capacity required to provide these upstream precursors. The absence of these amino acid and lipid precursors during the SSAF stage may limit the subsequent “Xunpei” phase, in which such compounds are required to drive the Maillard reaction that generates the characteristic color and smoky aroma of Shanxi aged vinegar.

Second, the multiphase medium differs. Huang et al. [20] analyzed the microbial community structure and metabolic functions of the vinegar SSAF microbiome and noted that microbial diversity and metabolic enzyme abundance in the solid-state environment markedly exceed those in liquid systems. The porous multiphase structure of industrial alcoholic fermentation provides a gas–liquid–solid interface that immobilizes extracellular enzymes and concentrates hydrophobic substrates for lipid hydrolysis and esterification. Chen et al. [21] emphasized the pivotal function of fungal mycelial networks as enzyme-substrate contact platforms in solid-state fermentation. The liquid-phase system compromises the synthesis and retention of long-chain hydrophobic esters, an effect attributable to aqueous dilution and to interfacial limitations.

Third, non-core microorganisms are absent. The trace flavor compounds distinctive to the IAF process, including higher fatty acid esters, ketones, furans and pyrazines, are likely derived from the secondary metabolism of non-core microbes or from interspecific transformations. Chen et al. [22] reviewed the contribution of actinomycetes to the SSAF microbiome, noting that non-LAB and non-yeast microbial groups, particularly actinomycetes and *Bacillus*, make important contributions to the generation of trace flavor compounds. Cheng et al. [23] summarized the interaction dynamics between LAB and yeasts in fermented foods and demonstrated that, beyond binary yeast–LAB interactions, third-party microorganisms can provide flavor complementarity through metabolic cross-feeding. In optimizing community structure to enhance the metabolic flux of major products, the SMC necessarily compromises the flavor complexity that arises from microbial diversity.

Aldehyde levels increased during the early phase of SMC fermentation, which may indicate activation of the Ehrlich pathway in yeast cells under the prevailing nitrogen-source conditions. The Ehrlich pathway is the primary route through which yeasts metabolize branched-chain amino acids, generating higher alcohols and the corresponding aldehydes. In the liquid SMC culture, the concentration of free amino acids in the medium may have been elevated, activating the Ehrlich pathway and leading to the accumulation of aldehyde intermediates. While aldehydes contribute to the overall aroma profile, excessive concentrations can produce an irritating, pungent sensation that negatively affects the sensory quality of vinegar. In traditional fermented grains, aldehyde accumulation is modulated by a gradual multi-enzyme cascade in which aldehyde dehydrogenase converts aldehydes to the corresponding organic acids and alcohol dehydrogenase reduces a proportion of aldehydes to alcohols. LAB and other non-yeast microorganisms also participate in aldehyde elimination through their aldehyde reductase systems. Zhang et al. [24] showed that *Pichia* spp. produce 2-phenylethanol and other volatile compounds via the Ehrlich pathway, and metatranscriptomic analysis has revealed regulatory features of this pathway in *Pichia*. The SMC, which lacks these regulatory components, underwent a shift in the aldehyde-alcohol-acid equilibrium. This observation suggests that future SMC designs should consider incorporating auxiliary strains with strong aldehyde dehydrogenase or aldehyde reductase activities to balance flavor harmony.

The three LAB strains exhibited distinct acid-production patterns: *L. plantarum* and *L. buchneri* exhibited peak acid concentrations at the earliest sampling point (7.32 g/L and 3.8 g/L at 24 h, respectively), indicative of a rapid-acidification type, whereas *L. amylovorus* demonstrated a progressive accumulation of acid, reaching 15.42 g/L at 168 h, suggesting a sustained-accumulation type. These disparities are closely related to the metabolic types of the strains: *L. plantarum* and *L. buchneri* are facultative heterofermentative LAB, whereas *L. amylovorus* is a homofermentative LAB with a higher monosaccharide-to-lactic acid conversion rate. The process of vinegar production in Shanxi involves a specific accumulation of lactic and acetic acids, which is crucial for the development of the distinctive “soft-sourness” profile of the final product. However, excessive acidity can impose severe stress on yeast viability. In the present study, the acid tolerance assays revealed that *S. cerevisiae* and *P. kudriavzevii* exhibited normal growth at acetic acid concentrations of ≤2 g/L. However, at concentrations ≥5 g/L, their growth was markedly inhibited.

Deng et al. [5] described a cooperative response mechanism of *P. kudriavzevii* and *S. cerevisiae* under lactic acid stress, with co-cultivation resulting in enhanced acid tolerance compared with monocultures. Chen et al. [8] further confirmed that fortified inoculation of indigenous lactobacilli in solid-state alcoholic fermentation, although promoting acid accumulation, did not significantly impair fermentation efficiency or yeast activity. This suggests the existence of acid-stress mutual adaptability between yeasts and LAB in adapted niches.

The acid-production curve of *L. amylovorus* continued to increase up to 168 h, indicating that the cumulative effect of acid stress on yeast viability is a critical parameter to monitor during extended SMC operation. Future optimization of the acid-alcohol-ester metabolic balance may be achieved by adjusting the inoculation ratios of the three LAB strains, for instance by reducing the initial proportion of *L. amylovorus* or by introducing lactate-utilizing microorganisms that act as a lactate sink.

The SMC accelerated the formation of several major volatile compounds in a liquid model system, but the following limitations should be acknowledged:

The liquid fermentation system used for the SMC differs fundamentally from the solid-state fermentation used in commercial SAV production in mass transfer efficiency, oxygen gradient distribution, and substrate heterogeneity. Therefore, the phenotypes observed in the liquid system cannot be directly extrapolated to actual solid-state scenarios.

The SMC contained solely yeasts and LAB and therefore lacked the *Bacillus* species and filamentous fungi that supply hydrolytic enzymes and modify flavor precursors in traditional *Jiupei*.

The present study was chiefly based on untargeted volatile flavor analysis and did not perform transcriptomic or proteomic validation of the underlying mechanisms; consequently, the gene-expression and metabolic-regulatory networks of individual strains under co-culture conditions remain unclear.

Strain-specific population dynamics were not monitored during SMC fermentation. Whether all six strains remained viable and metabolically active throughout the 7-day period, and whether individual strains became dominant, therefore remains unknown; the metabolic output reported here is attributed to the consortium as a whole and cannot be assigned to individual members.

The experimental design did not include monocultures of the individual strains or simplified yeast–LAB combinations. The present data consequently do not demonstrate synergistic metabolism, and the magnitude of any interaction effect relative to the additive contributions of the six strains remains to be established.

Volatile compounds were quantified semi-quantitatively as 2-octanol equivalents and identified by mass spectral and retention index matching without confirmation using authentic standards. Reported concentrations are therefore not absolute, identification of individual compounds is tentative, and no odor activity values, aroma thresholds, GC-olfactometry or sensory data were generated; the volatile profiles are consequently interpreted as chemical rather than as sensory evidence.

The IAF samples were obtained from a single industrial production campaign, so batch-to-batch variation in the industrial process was not captured. The comparison with the SMC should therefore be regarded as a comparison against one representative production run.

Modular extension of the present consortium is supported by several lines of evidence. Wang and Xu [25] showed that microbial succession and targeted metabolic modules can restructure volatile profiles during the fermentation of Chinese light aroma-style liquor, and Zhang et al. [26] reviewed strategies and challenges of microbiota regulation in Baijiu brewing, outlining a “core microbiota plus auxiliary strain” modular design. Li and Liu [27] summarized the engineering design principles and regulatory strategies available for synthetic microbial consortia, including quorum-sensing-based control and metabolic-flux redirection, which offer routes toward stabilizing defined communities. Translating such approaches to the SAV alcoholic fermentation stage will first require validation of the six-strain consortium in an authentic solid-state matrix.

**Figure 5:**
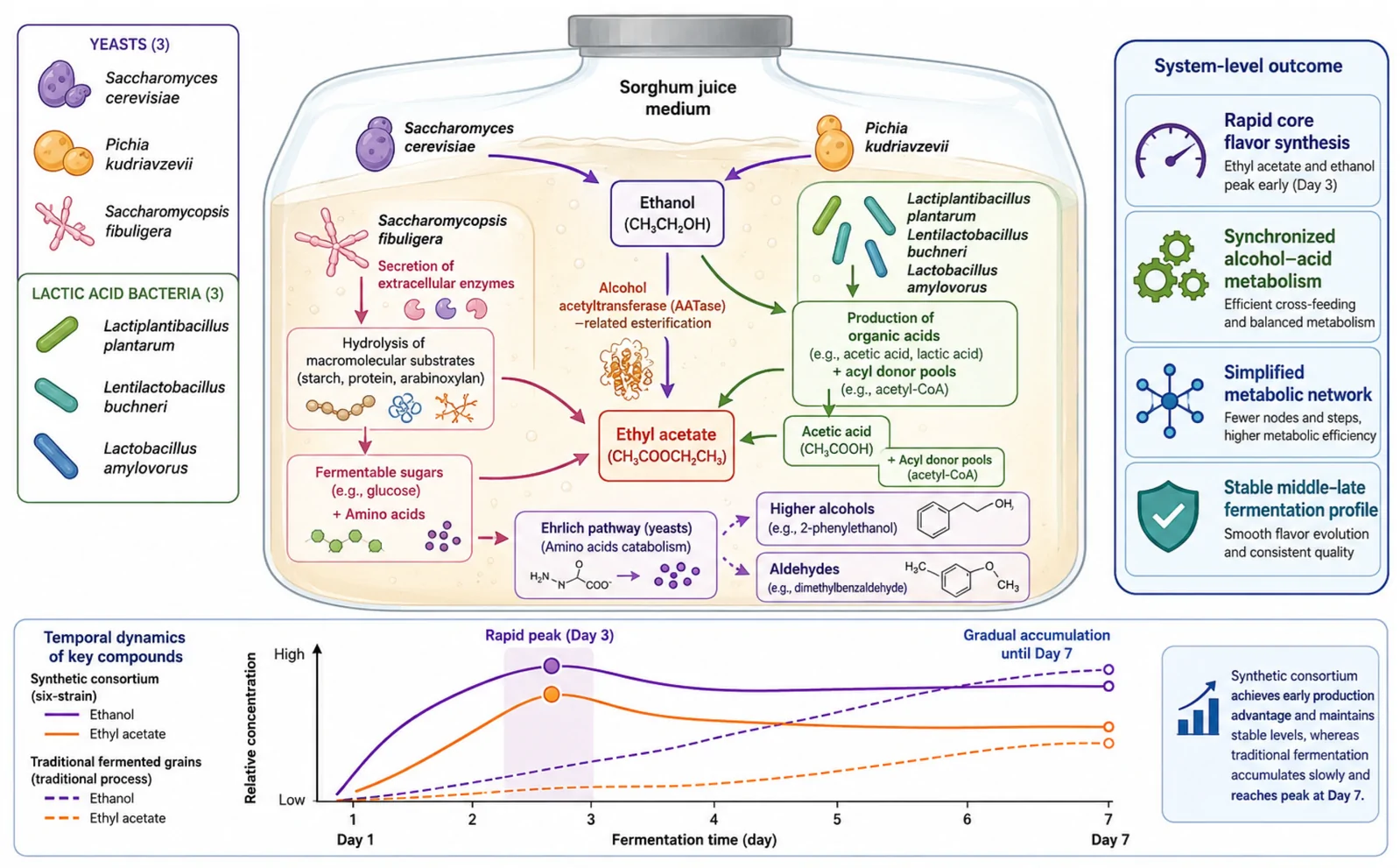
The fermentation mechanism of synthetic microbial consortia.

In light of the research findings, a synthetic microbial consotia fermentation mechanism is hereby proposed, as illustrated in Figure 5. Future studies should evaluate the performance of the six-strain SMC in authentic solid-state SAV fermentation matrices and monitor strain-specific population dynamics throughout fermentation, so that the persistence and metabolic activity of each member can be established. Monoculture and simplified yeast–LAB combination controls should be included to distinguish interspecific interactions from additive single-strain metabolism. Key volatile compounds should be confirmed with authentic standards and, where possible, complemented by odor activity values or GC-olfactometry so that sensory relevance can be demonstrated. Additional auxiliary strains with demonstrated safety and relevant hydrolytic or aroma-related functions may subsequently be evaluated to increase metabolic diversity, and metatranscriptomic and metabolomic approaches could help to clarify interspecies metabolic interactions and to guide optimization of consortium composition and inoculation ratios.

## 5. Conclusions

This study shows that a six-strain synthetic microbial consortium assembled from isolates recovered from *Daqu* and *Jiupei* reproduced selected metabolic features of Shanxi aged vinegar (SAV) alcoholic fermentation in a liquid model system, and that several major volatile compounds reached their maximum concentrations earlier than in the industrial system examined. These observations do not imply that the consortium reproduces or outperforms industrial solid-state fermentation: the two systems differ in substrate composition, physical structure and mass transfer, and the volatile data are semi-quantitative and based on tentative identifications. Within these limits, the consortium provides a controllable experimental platform for examining how community composition shapes volatile output. Its reduced volatile diversity relative to the industrial system indicates that the complexity of the traditional process is not readily recapitulated by a six-member community and underscores the value of defined consortia as analytical tools for dissecting, rather than replacing, traditional fermentation.

## Figures and Tables

**Table 1:** Identification and condensed morphology of microbial strains isolated from *Daqu* and *Jiupei*.

Source	Strain Code	Identification Result	Key Characteristics
Daqu	DQ-2	*Saccharomycopsis fibuligera*	White velvety circular colonies; round to ovoid budding yeast cells
	DQ-5	*Aspergillus flavus*	Irregular green-yellowish mycelium; globose conidia
	DQ-6	*Saccharomyces cerevisiae*	Smooth milky white colonies; ovoid budding yeast cells
	DQ-10	*Pichia kudriavzevii*	Smooth yellowish-brown colonies; ellipsoidal yeast cells
Jiupei	JP-08	*Lactiplantibacillus plantarum*	Circular smooth milky white colonies; Gram-positive short rods
	JP-10	*Lentilactobacillus buchneri*	Subcircular smooth milky white colonies; Gram-positive short rods
	JP-90	*Ligilactobacillus amylovorus*	White colonies with Ca^2+^ dissolution zone; Gram-positive short rods
	JP-56	*Levilactobacillus brevis*	Milky white colonies with Ca^2+^ dissolution zone; Gram-positive short rods
	JP-68	*Limosilactobacillus fermentum*	Creamy yellow circular colonies; Gram-positive ellipsoidal rods
	JP-22	*Bacillus pumilus*	Circular moist white colonies; Gram-positive long rods
	JP-23	*Bacillus licheniformis*	Dry inconspicuous colonies; Gram-positive long rods
	JP-30	*Bacillus* sp.	Large irregular rough colonies; Gram-positive spore-forming rods
	JP-32	*Bacillus megaterium*	Circular smooth white colonies; Gram-positive long rods
	JP-33	*Bacillus subtilis*	Raised dry irregular colonies; Gram-positive long rods
	JP-35	*Pantoea agglomerans*	Creamy yellow circular colonies; Gram-positive short rods
	JP-36	*Fructilactobacillus fructivorans*	Milky white circular colonies; Gram-positive ellipsoidal rods
	JP-40	*Bacillus cereus*	Mucilaginous colonies; Gram-positive ellipsoidal rods
	JP-41	*Lysinibacillus* sp.	Gram-positive spore-forming long rods
	JP-42	*Bacillus velezensis*	Milky white irregular colonies; Gram-positive spore-forming rods
	JP-45	*Bacillus amyloliquefaciens*	White moist irregular colonies; Gram-positive rods

## Data Availability

The original contributions presented in the study are included in the article. The 16S rRNA gene and ITS sequences generated in this study have been deposited in GenBank under the accession numbers listed in Supplementary Table S2; further inquiries can be directed to the corresponding author.

## Supplementary Materials

The following supporting information can be downloaded at: https://www.mdpi.com/article/10.3390/foods15183286/s1, Table S1. Identification and condensed morphology of microbial strains isolated from *Daqu* and *Jiupei*. Table S2. Table of Accession Numbers and Corresponding Species. Table S3. Retention time and similarity of key volatile compounds. Table S4. Process parameters of the industrial alcoholic fermentation (IAF) of Shanxi aged vinegar.

## Abbreviations

The following abbreviations are used in this manuscript:

SAV	Shanxi aged vinegar
SMC	Synthetic Microbial Consortium
SSF	solid-state fermentation
AF	alcoholic fermentation
LAB	lactic acid bacteria
IAF	industrial alcoholic fermentation of fermented grains
HS-SPME	headspace solid-phase microextraction
ANOVA	analysis of variance
CFU	colony-forming unit
GC-MS	gas chromatography–mass spectrometry
OD600	optical density at 600 nm
PCA	principal component analysis
RI	retention index
SPME	solid-phase microextraction
