# Supplementary material for: Directional Construction of a Synthetic Microbial Consortium for Shanxi Aged Vinegar: Metabolic Kinetics and Accelerated Formation of Major Volatile Compounds
Source: Foods. 2026 Sep 17;15(18):3286. doi: 10.3390/foods15183286 (PMC13606686; doi:10.3390/foods15183286)
Supplement: Supplementary file 1 [file foods-15-03286-s001.zip › Supplementary Materials Table S1.pdf]

Table S1. Identification and condensed morphology of microbial strains isolated from *Daqu* and *Jiupei*.

Source	Strain code	Morphological characteristics	Microscopic morphology	Identification result
Daqu	DQ-2	Circular, 5 mm, white margin, raised center, white velvety, firmly attached.	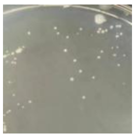	*Saccharomycopsis fibuligera*
		Budding cells, round to ovoid.	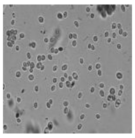	
Daqu	DQ-5	Irregular, 25 mm, mycelium present.	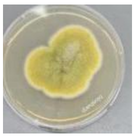	*Aspergillus flavus*
		Reticulate, conidia spherical.	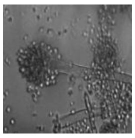	
Daqu	DQ-6	30 mm diameter, white margin, milky white, smooth surface.	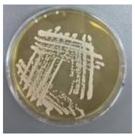	*Saccharomyces cerevisiae*
		Ovoid, budding.	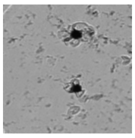	
Daqu	DQ-10	28 mm diameter, smooth surface, entire margin, initially white turning yellowish-brown.	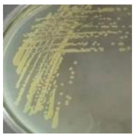	*Pichia kudriavzevii*
		25 mm diameter, ellipsoidal.	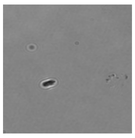	

Source	Strain code	Morphological characteristics	Microscopic morphology	Identification result
Jiupei	JP-08	Milky white, circular, smooth, entire margin.	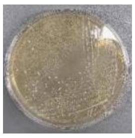	*Lactiplantibacillus plantarum*
		Short rods, Gram-positive.	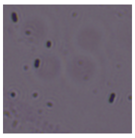	
Jiupei	JP-10	Milky white, subcircular, smooth, entire margin.	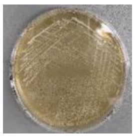	*Lentilactobacillus buchneri*
		Short rods, Gram-positive.	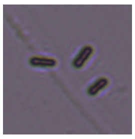	
Jiupei	JP-90	Circular, 1 mm, white, Ca^{2+} dissolution zone present.	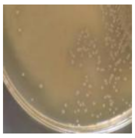	*Ligilactobacillus amylovorus*
		Short rods, Gram-positive.	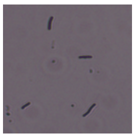	
Jiupei	JP-56	Circular, 1 mm, milky white, Ca^{2+} dissolution zone present.	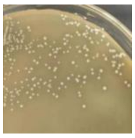	*Levilactobacillus brevis*
		Short rods, Gram-positive.	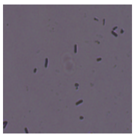	
Jiupei	JP-68	Circular, 1 mm, creamy yellow.	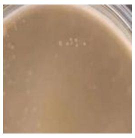	*Limosilactobacillus fermentum*

**Source**	**Strain code**	**Morphological characteristics**	**Microscopic morphology**	**Identification result**
		Ellipsoidal, Gram-positive.	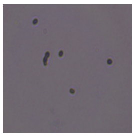	
Jiupei	JP-22	Circular, 2 mm, milky white, moist surface.	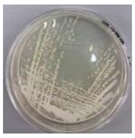	*Bacillus pumilus*
		Long rods, Gram-positive.	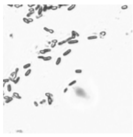	
Jiupei	JP-23	Dry surface, colonies inconspicuous.	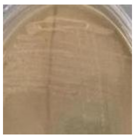	*Bacillus licheniformis*
		Long rods, Gram-positive.	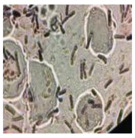	
Jiupei	JP-30	Large, irregular colony, rough surface.	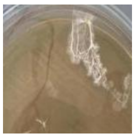	*Bacillus sp.*
		Long rods, spore-forming, Gram-positive.	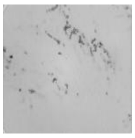	
Jiupei	JP-32	Circular, 1 mm, white, relatively smooth.	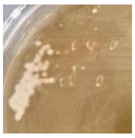	*Bacillus megaterium*
		Long rods, Gram-positive.	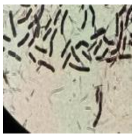	

Source	Strain code	Morphological characteristics	Microscopic morphology	Identification result
Jiupei	JP-33	Raised, dry surface, 5 mm, irregular margin.<br>Long rods, Gram-positive.	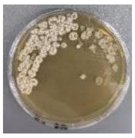<br>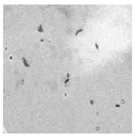	*Bacillus subtilis*
Jiupei	JP-35	Rod-shaped, Gram-positive.<br>The colonies are round, 1 mm in diameter, and creamy yellow in color.	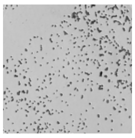<br>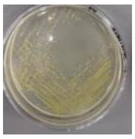	*Pantoea agglomerans*
Jiupei	JP-36	Ellipsoidal, Gram-positive.<br>The colonies are round, 2 mm in diameter, and milky white.	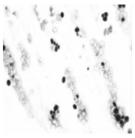<br>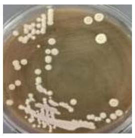	*Fructilactobacillus fructivorans*
Jiupei	JP-40	Ellipsoidal, occurring singly or in pairs, Gram-positive.<br>The colonies are mucilaginous, with relatively well-defined edges.	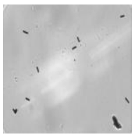<br>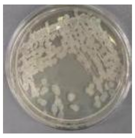	*Bacillus cereus*
Jiupei	JP-41	Long rod-shaped, with visible spores, Gram-positive	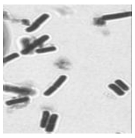	*Lysinibacillus*

Source	Strain code	Morphological characteristics	Microscopic morphology	Identification result
		Long rod-shaped with spores	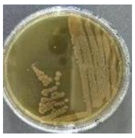	
Jiupei	JP-42	Long, rod-shaped with spores<br><br>The colonies are 2 mm in diameter, milky white, with irregular edges and a relatively smooth surface.	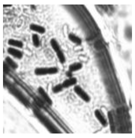<br>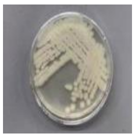	*Bacillus velezensis*
Jiupei	JP-45	Rod-shaped, Gram-positive.<br><br>The colonies are irregularly circular, 6 mm in diameter, white, and have a moist	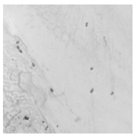<br>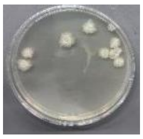	*Bacillus amyloliquefaciens*
