# Supplementary material for: Directional Construction of a Synthetic Microbial Consortium for Shanxi Aged Vinegar: Metabolic Kinetics and Accelerated Formation of Major Volatile Compounds
Source: Foods. 2026 Sep 17;15(18):3286. doi: 10.3390/foods15183286 (PMC13606686; doi:10.3390/foods15183286)
Supplement: Supplementary file 1 [file foods-15-03286-s001.zip › Supplementary Materials Table S2.pdf]

Table S2. Table of accession numbers and corresponding species

Accession Number	Species Name
MZ165223.1	*Bacillus subtilis*
OP984784.1	*Bacillus subtilis*
JF899258.1	*Bacillus amyloliquefaciens subsp. plantarum*
OR206161.1	*Bacillus velezensis*
MT834617.1	*Bacillus zhangzhouensis*
MN704531.1	*Bacillus pumilus*
OR975555.1	*Bacillus cereus*
HQ840732.1	*Bacillus megaterium*
PP237225.1	*Pantoea agglomerans*
KR816183.1	*Lactobacillus fermentum*
OM758301.1	*Lactiplantibacillus plantarum*
MG461978.1	*Lactobacillus parabuchneri*
AB268535.1	*Lactobacillus brevis*
MW020293.1	*Lactobacillus amylovorus*
MH244419.1	*Aspergillus oryzae*
KP132509.1	*Pichia kudriavzevii*
KC544488.1	*Saccharomyces cerevisiae*
KY767619.1	*Saccharomycopsis fibuligera*
