# Supplementary material for: Directional Construction of a Synthetic Microbial Consortium for Shanxi Aged Vinegar: Metabolic Kinetics and Accelerated Formation of Major Volatile Compounds
Source: Foods. 2026 Sep 17;15(18):3286. doi: 10.3390/foods15183286 (PMC13606686; doi:10.3390/foods15183286)
Supplement: Supplementary file 1 [file foods-15-03286-s001.zip › Supplementary Materials Table S3.pdf]

Table S3. Retention time and similarity of key volatile compounds

Compound	CAS No.	Retention time (min)	Similarity (%)
Ethanol	64-17-5	1.305	97
Ethyl acetate	141-78-6	1.937	98
2-Phenylethanol (β-Phenethyl alcohol)	60-12-8	29.140	98
2,4-Dimethylbenzaldehyde	15764-16-6	24.262	96
3,5-Dimethylbenzaldehyde	5779-95-3	23.713	90
m-Xylene	108-38-3	7.353	98
Ethyl lactate	687-47-8	5.410	98
Diethyl succinate	123-25-1	22.868	98
Ethyl hexadecanoate (Ethyl palmitate)	628-97-7	34.035	96
Ethyl propionate	105-37-3	2.875	94
2,4-Di-tert-butylphenol	96-76-4	29.103	97
3-Methyl-1-butanol	123-51-3	3.542	97
Nonanal	124-19-6	18.001	94
Ethyl phenylacetate	101-97-3	25.171	85
Ethyl octanoate	106-32-1	23.796	95
