# Supplementary material for: Directional Construction of a Synthetic Microbial Consortium for Shanxi Aged Vinegar: Metabolic Kinetics and Accelerated Formation of Major Volatile Compounds
Source: Foods. 2026 Sep 17;15(18):3286. doi: 10.3390/foods15183286 (PMC13606686; doi:10.3390/foods15183286)
Supplement: Supplementary file 1 [file foods-15-03286-s001.zip › Supplementary Materials Table S4.pdf]

Table S4. Process parameters of the industrial alcoholic fermentation (IAF) of Shanxi aged vinegar.

Parameter	Specification
Sorghum-to-water ratio and adjuncts (%, w/w)	Sorghum as the principal substrate, with barley and wheat bran as adjuncts[a]. Sorghum-to-water ratio: [to be confirmed][c] (%, w/w); adjuncts: [to be confirmed][c] (%, w/w). Reported values for the traditional process: 60–65 kg water added before steaming, 215–225 kg after steaming and 65 kg before vatting, per 100 kg sorghum (total water approximately 300–340%, w/w)[b].
Daqu addition rate (%, w/w) and viable count of the starter (CFU/g)	Daqu addition rate: [to be confirmed][c] (%, w/w); the traditional process adds 62.5 kg of ground Daqu per 100 kg sorghum (62.5%, w/w)[b]. Viable count of the starter at inoculation: [to be confirmed][c] (CFU/g).
Temperature profile and peak temperature (°C)	Temperature profile: [to be confirmed][c]. Reported values for the traditional process: the mash is charged at 20–25 °C, rises to approximately 30 °C on day 3 and reaches a peak of approximately 34 °C on day 4, after which it declines, with post-fermentation held at 18–20 °C[b]. Peak temperature: approximately 34 °C[b].
Total duration (days) and turning/aeration regime	Total duration: [to be confirmed][c] (days). Turning/aeration regime: [to be confirmed][c]; the traditional process is turned twice daily during the first 3 days (open, aerobic fermentation) and sealed for anaerobic post-fermentation from day 4 onwards, giving a total duration of approximately 18–21 days[b].
Vessel type, batch size and initial moisture content (%, w/w)	Vessel type: [to be confirmed][c]; batch size: [to be confirmed][c]; initial moisture content: [to be confirmed][c] (%, w/w).

[a] Derived from the manuscript: the raw materials of the industrial alcoholic fermentation are described as sorghum, barley and wheat bran (Sections 2 and 4).

[b] Reported values for the traditional Shanxi aged vinegar alcoholic fermentation process (Ministry of Commerce “Time-honoured Brand Digital Museum”, Baoyuan Laocufang traditional brewing technique; Food Nutrition, “Production of Shanxi aged vinegar”). These are reference values for the traditional process and must be verified against the actual production protocol before submission.

[c] Not specified in the manuscript or in the consulted sources. To be supplied by the authors from plant production records.
